# Complete Genome Sequence of *Aneurinibacillus migulanus* E1, a Gramicidin S- and d-Phenylalanyl-l-Propyl Diketopiperazine-Deficient Mutant

**DOI:** 10.1128/genomeA.01441-15

**Published:** 2015-12-17

**Authors:** Lassaad Belbahri, Faizah N. Alenezi, Lenka Luptakova, Mostafa E. Rateb, Steve Woodward

**Affiliations:** a Laboratory of Soil Biology, University of Neuchatel, Neuchâtel, Switzerland; b NextBiotech, Agareb, Tunisia; c University of Aberdeen, Institute of Biological and Environment Science, Aberdeen, Scotland, United Kingdom; d University of Veterinary Med & Pharmacy, Institute of Biology, Zoology & Radiobiology, Department of Biology and Genetics, Košice, Slovakia; e School of Science & Sport, University of West of Scotland, Paisley, United Kingdom

## Abstract

We report here the complete genome sequence of the *Aneurinibacillus migulanus* E1 mutant deficient in gramicidin S (GS) and d-phenylalanyl-l-propyl diketopiperazine (DKP) formation. The genome consists of a circular chromosome (6,301,904 bp, 43.20% G+C content) without any plasmid. The complete genome sequence enables further investigation of the biosynthetic mechanism and the biological function of gramicidin S.

## GENOME ANNOUNCEMENT

Gramicidin S (GS) is a cationic cyclic decapeptide with the primary structure [cyclo-(Val-Orn-Leu-d-Phe-Pro)_2_] (1). GS is an extremely powerful antibiotic drug against a broad spectrum of both Gram-negative and Gram-positive bacteria and against several pathogenic fungi (2–5). GS-deficient mutants in *Aneurinibacillus migulanus* have been classified into five different categories. The mutant E1 belongs to the fifth group that contains both a phenylalanine-activating enzyme and a complex of proline-, valine-, ornithine-, and leucine-activating enzymes similar to those of the wild-type strain (6). E1 is unable to synthetize GS or d-phenylalanyl-l-propyl diketopiperazine (DKP).

The genome of the *A. migulanus* E1 mutant was sequenced by the Illumina HiSeq 2000 platform (2 × 125 bp), and the sequencing coverage was 100×. After sequencing, the reads were assembled using CLC Genomics Workbench 7.0.3 (CLC bio). Annotation was performed using the NCBI Prokaryotic Genomes Automatic Annotation Pipeline (PGAAP) version 2.9 (7). The whole genome is represented by a circular chromosome of 6,301,904 bp with no plasmid. The G+C content was around 43.20%, as reported for other *Aneurinibacillus* species (2, 3). The genome contains 5,672 coding sequences (CDSs), 11 rRNA operons, and 95 tRNA genes. Using antiSMASH 3.0, 12 gene clusters for secondary metabolites have been predicted in the genome of the E1 *A. migulanus* mutant strain (8). Nonribosomal peptides, terpenes, polyketides, siderophores, bacteriocins, microcins, lasso peptides, and aryl-polyene are among the predicted secondary metabolites. This suggests an important structural diversity of E1 secondary metabolites. The availability of the genome sequence of the E1 mutant is supposed to help identify mutations in this gramicidin S-deficient mutant and therefore to understand the genetic regulation of gramicidin S biosynthesis in *A. migulanus.*

### Nucleotide sequence accession number.

The sequence of the E1 chromosome has been deposited in GenBank under accession the no. LIXL00000000. The version described in this paper is the first version.

## References

[1] Marahiel MA, Danders W, Krause M, Kleinkauf H 1979 Biological role of gramicidin S in spore functions. Studies on gramicidin-S-negative mutants of *Bacillus brevis* ATCC 9999. Eur J Biochem 99:49–55. doi:10.1111/j.1432-1033.1979.tb13229.x. 90616

[2] Alenezi FN, Weitz HJ, Belbahri L, Nidhal J, Luptakova L, Jaspars M, Woodward S 2015 Draft genome sequence of *Aneurinibacillus migulanus* NCTC 7096. Genome Announc 3(2):e00234-15. doi:10.1128/genomeA.00234-15. 25838489 PMC4384493

[3] Alenezi FN, Weitz HJ, Belbahri L, Ben Rebah H, Luptakova L, Jaspars M, Woodward S 2015 Draft genome sequence of *Aneurinibacillus migulanus* strain Nagano. Genome Announc 3(2):e00232-15. doi:10.1128/genomeA.00232-15. 25838487 PMC4384491

[4] Alenezi FN, Fraser S, Bełka M, Doğmuş TH, Hečkova Z, Oskay F, Belbahri L, Woodward S 2015 Biological control of Dothistroma needle blight on pine with *Aneurinibacillus migulanus*. Forest Pathol, in press. doi:10.1111/efp.12237.

[5] Alenezi FN, Rekik I, Bełka M, Ibrahim AF, Luptakova L, Jaspers M, Woodward S, Belbahri L 2015 Strain-level diversity of secondary metabolism in the biocontrol species *Aneurinibacillus migulanus*. Microbiol Res 182:116–124. doi:10.1016/j.micres.2015.10.007. 26686620

[6] Shimura K, Iwaki M, Kanda M, Hori K, Kaji E, Hasegawa S, Saito Y 1974 On the enzyme system obtained from some mutants of *Bacillus brevis* deficient in gramicidin S formation. Biochim Biophys Acta 338:577–587. doi:10.1016/0304-4165(74)90321-3.

[7] Angiuoli SV, Gussman A, Klimke W, Cochrane G, Field D, Garrity GM, Kodira CD, Kyrpides N, Madupu R, Markowitz V, Tatusova T, Thomson N, White O 2008 Toward an online repository of Standard Operating Procedures (SOPs) for (meta)genomic annotation. Omics 12:137–141. doi:10.1089/omi.2008.0017. 18416670 PMC3196215

[8] Weber T, Blin K, Duddela S, Krug D, Kim HU, Bruccoleri R, Lee SY, Fischbach MA, Müller R, Wohlleben W, Breitling R, Takano E, Medema MH 2015 antiSMASH 3.0—a comprehensive resource for the genome mining of biosynthetic gene clusters. Nucleic Acids Res 43:W237–W243. doi:10.1093/nar/gkv437. 25948579 PMC4489286

